# A Case of Parvovirus-Related Haemophagocytic Lymphohistiocytosis in a Patient with HbH Disease

**DOI:** 10.1155/2018/8057045

**Published:** 2018-12-24

**Authors:** Veeraraghavan Meyyur Aravamudan, Chaozer Er, Ikram Hussain, Nicholas wong wai Cheong, Chong Chern Hao, Navin Kuthah, Emily En-Xian Tan, Bingwen Eugene Fan

**Affiliations:** ^1^Department of Medicine, Woodlands Health Campus (WHC), Singapore; ^2^Department of Haematology, Tan Tock Seng Hospital, Singapore

## Abstract

Hemophagocytic lymphohistiocytosis (HLH) is rare and life-threatening medical emergency. Parvovirus infection is rarely associated with HLH. We report a case of parvovirus-related HLH in a patient with alpha thalassaemia (HbH disease). The patient responded well to a course of dexamethasone without the need of etoposide. Based on our literature search, this is the first case of parvovirus related HLH in a patient with HbH disease in the medical literature.

## 1. Introduction

Hemophagocytic lymphohistiocytosis (HLH) is rare and life-threatening medical emergency [1]. Our patient is a middle-aged Chinese lady with alpha thalassaemia (HbH disease), presented to us with persistent fever and rapidly progressive pancytopaenia. She was eventually diagnosed with HLH triggered by parvovirus infection. She responded well to a course of high-dose steroid. To our knowledge, this is the first case of parvovirus-associated HLH in a patient with HbH disease.

## 2. Case Report

Mdm A, a 36-year-old Chinese lady with alpha thalassaemia (HbH disease), presented to us with a one-week history of fever and chills. There was no localizing symptom of infection. Her physical examination findings were unremarkable.

Initial investigation (Table 1) revealed pancytopaenia: hemoglobin (Hb) 7.3 g/dL (11–14.7 g/dL), white cell count (WC) 1.68 × 10^9^/L (3.37 to 8.38 × 10^9^/L), and platelet count 92 × 10^9^/L (172–378 × 10^9^/L). C-reactive protein was 24 while procalcitonin was 0.14. Renal function, liver enzymes, chest X-ray, and urinalysis were unremarkable.

She was given piperacillin-tazobactam empirically. Despite antibiotic, she remained febrile on day 3 of admission. Three sets of blood culture and urine culture were negative. Dengue NS 1 antigen was negative. Piperacillin-tazobactam was changed to carbapenem.

**Figure 1:**
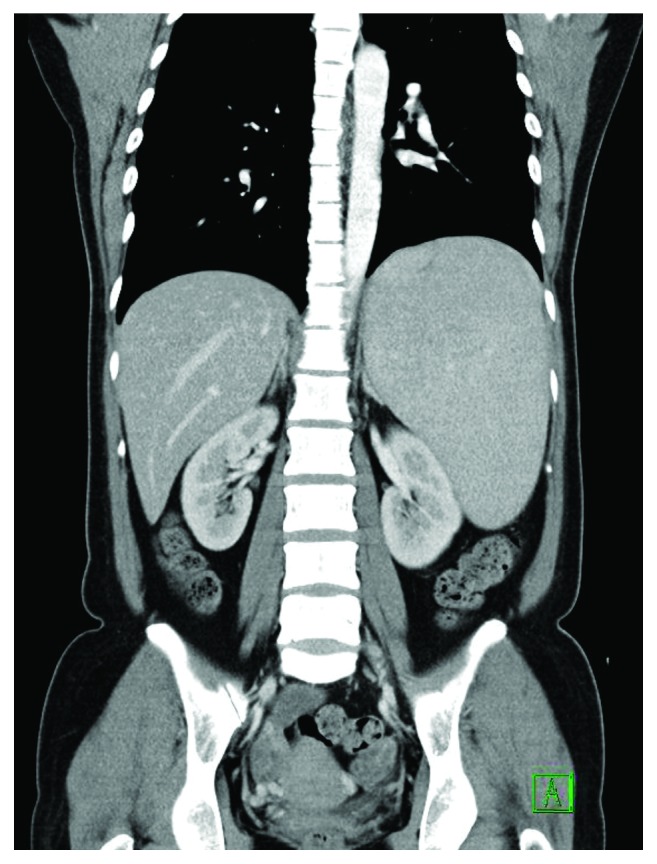
Computed tomographic coronal section showing hepatosplenomegaly.

Computed tomography (CT) of the abdomen and pelvis was arranged to investigate the cause of fever. It showed hepatosplenomegaly but no intra-abdominal abscesses (Figure 1).

**Figure 2:**
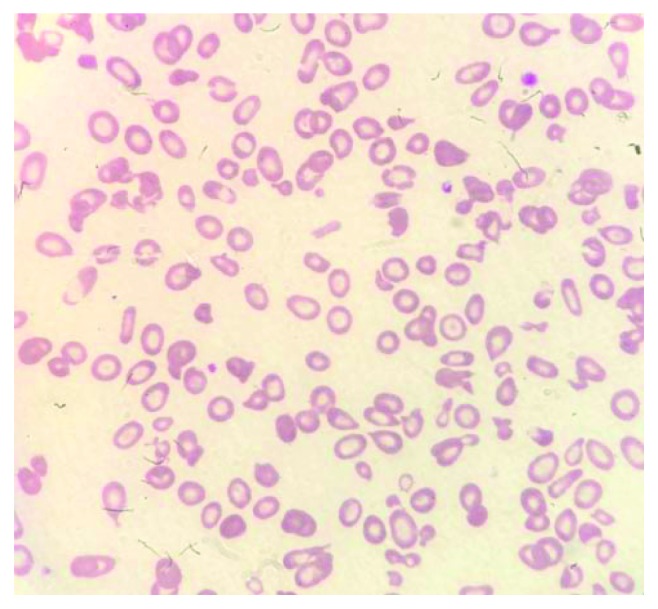
Marked anisopoikilocytosis with microcytosis. Teardrop cells, target cells, and contracted red blood cells can be seen. Leukopenia and thrombocytopenia are present.

**Figure 3:**
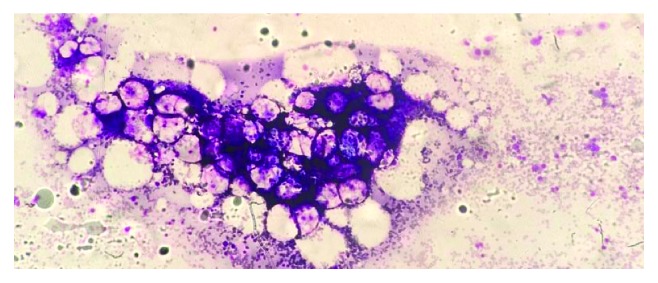
Hypocellular marrow fragment for age with decreased cell trails.

**Figure 4:**
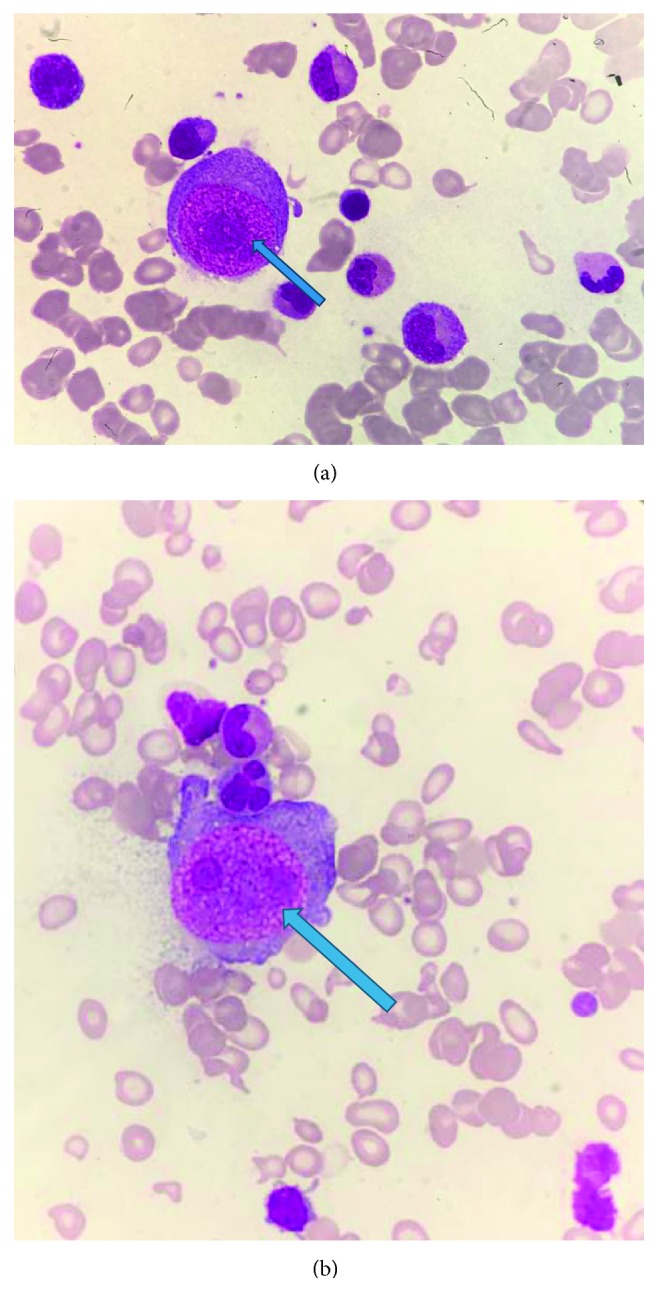
(a, b) Giant erythroblasts with inclusion bodies dwarfing the granulocyte precursors.

**Figure 5:**
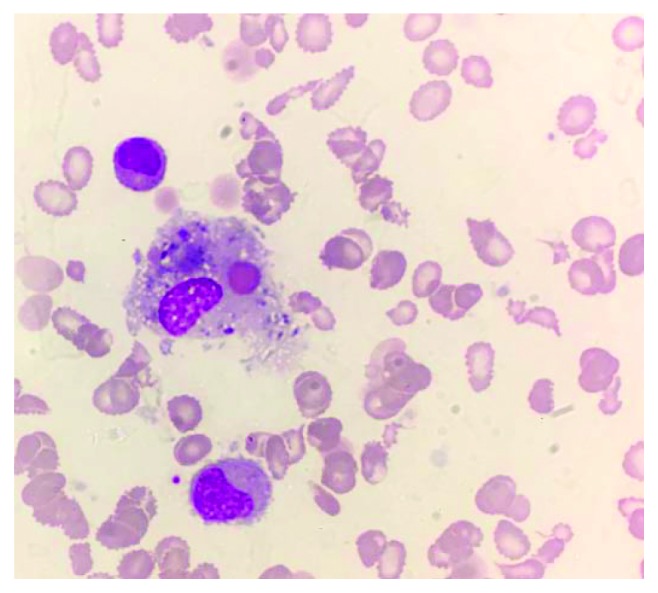
Haemosiderin-laden macrophage with active haemophagocytosis of an RBC.

The pancytopaenia progressively worsened (Table 2). Peripheral blood film showed marked leukopenia, thrombocytopenia, and significant anisopoikilocytosis with microcytosis and target and tear drop cells consistent with a picture of thalassaemia intermedia (Figure 2). A bone marrow examination was therefore performed. Typical bone marrow finding in a patient with HbH disease alone would reveal a hypercellular marrow with erythroid hyperplasia and marked dyserythropoiesis. Mdm. A's marrow however was hypocellular with decreased cell trails on the aspirate (Figure 3). There was marked erythroid hypoplasia with rare giant erythroblasts, and inclusion bodies were seen (Figure 4). There were also increased numbers of macrophages with active haemophagocytosis (Figure 5). There was no evidence of lymphoma on bone marrow examination. Haemophagocytosis is however not exclusive of HLH. It may also be present in critically ill patients. Mdm. A was diagnosed with HLH based on the fact that she fulfilled 5 out of the 9 diagnostic criteria of HLH used in the HLH 2004 trial: fever >38.5°C, splenomegaly, peripheral blood cytopaenia, haemophagocytosis in marrow, and ferritin >500 ng/ml.

She was then transferred to a tertiary hospital for further treatment. 16 mg of dexamethasone once daily was initiated. Dexamethasone dose was reduced by 50% every 5 days. After 5 days of 2 mg once daily dexamethasone, the steroid was stopped. She responded well to the 20-day course of dexamethasone. She was reviewed in the clinic once a week after discharge, and other than her baseline anaemia due to HbH disease, other cell lines have recovered. Parvovirus B19 PCR was noted to be positive during her clinic visit. Diagnosis of parvovirus-induced HLH was made. She remained well in the subsequent clinic follow-up.

## 3. Discussion

HLH is a syndrome caused by increased immune response, excessive macrophage activation, and cytokine release that leads to multiorgan dysfunction [1, 2]. It can be primary (familial) or secondary (reactive) [1]. Primary HLH is due to genetic defect and is mainly seen in children [1]. Secondary HLH occurs in adults [1]. It can be triggered by infection, autoimmune disease, or malignancy [1]. Infection-associated HLH (IAHLH) is commonly associated with Epstein–Barr virus (EBV) and cytomegalovirus (CMV) [3]. Klebsiella, HIV, herpes simplex, adenovirus, and parasitic and fungal infections have been implicated [3]. A study in Japan found that 1% of IAHLH was caused by parvovirus [1].

Diagnosis of HLH is challenging as the clinical features are nonspecific and can be seen in other inflammatory conditions such as sepsis and malignancy [1]. Based on the HLH 2004 guideline, 5 out of the 8 clinical criteria must be met for the diagnosis to be made [1]. They are as follows [1]:

- Fever >38.5°C
- Splenomegaly
- Cytopaenia (at least 2 cell lineages are affected)
- Hypertriglyceridaemia
- Ferritin >500 ng/ml
- Haemophagocytosis in bone marrow, spleen, or lymph nodes
- Low or absent natural killer (NK) cell activity
- Elevated interleukin (IL) 2 receptor

The last two markers mentioned above are not available in Singapore. A ferritin level of >10,000 ng/L was reported to have 90% sensitivity and 96% specificity for HLH [1]. A higher ferritin level was reported to reflect poorer prognosis and is therefore used as a marker to monitor treatment [1]. Haemophagocytosis in the marrow is not specific for HLH [1]. It can also be seen in haematologic malignancies or EBV infection [1]. Haemophagocytosis can be absent in HLH [1, 4]. Its sensitivity is 80–83%, and specificity is 60% [1].

Kio et al. described a case of sickle cell crisis associated with HLH [5]. The patient recovered without immunosuppression [5]. They mentioned that some cytokines implicated in HLH like IL-6, IL-2R, and TNF-alpha are raised in sickle cell disease [5]. Moreover, zinc deficiency which is common in sickle cell disease and thalassaemia major is related to impaired NK-cell activity and HLH pathogenesis [5]. We however found no prior report on HLH in HbH disease. We believe that the same pathogenesis applied in our case.

HLH treatment consists of dexamethasone and etoposide [1]. Our patient responded well to a 20-day course of dexamethasone without etoposide. Yuan et al. [1] have also reported a case of B19-related IAHLH that responded well to dexamethasone monotherapy previously. Immunoglobulin has been used with success in some cases [2, 6].

HLH is fatal if left untreated [1]. Median survival of <2 months and mortality of 58–75% were described [1]. EBV-associated IAHLH carries high mortality while B19 infection related IAHLH has better prognosis [2]. Hereditary spherocytosis is the most common underlying disease in B19-associated HLH [2]. We believe that HbH, being a chronic haemolytic anaemia, may also have increased susceptibility for B19 infection. It appears important to perform an extensive workup for the infectious aetiology of HLH as management and prognosis may be different [2].

In conclusion, HLH is a life-threatening and complex clinical syndrome that has nonspecific clinical findings. High degree of suspicion is required for early diagnosis. Prompt treatment of the underlying aetiology combined with immunosuppressant seems to be ideal treatment. HbH, being a haemolytic anaemia, may have increased risk of getting B19-related HLH. More prospective studies are required to identify the treatment and outcome of B19-related HLH.

## Figures and Tables

**Table 1:** Values of relevant laboratory tests on presentation.

Test	Results	Unit	Reference interval
White blood cell	1.68	×10^9^/L	3.40–9.60
Hemoglobin	7.3	g/dL	11.5–15.1
Mean cell volume	58.4	fL	80.0–95.0
Mean corpuscular hemoglobin concentration	18.6	g/dL	32.0–36.0
Platelets	92		132–372
Creatinine	56	mg/dL	0.6–1.0
Urea	2.5	mg/dL	5.6–18.2
Albumin	47	g/dL	3.8–4.8
Bilirubin, total	31	mg/dL	0.3–1.8
Aspartae aminotransferase	41	U/L	11–34
Alanine aminotransferase	19	U/L	10–36
Alkaline phosphatase	43	U/L	40–130
Prothrombin time	11.5	Seconds	9.2–11.4
Beta-hcg (serum)	Negative		
Vitamin B12	363	Nmol/l	7–37
Folate	33	Pmol/l	145–569
Serum iron	37.7	Umol/Litre	6.6–30.4
Ferritin	944	Ng/ml	13–150
Iron saturation	84		15–150
Fibrinogen	3.06	g\L	1.8–3.5
Total iron binding capacity	45	Umol/Litre	52–94
Haptoglobin	38	mg/dL	30–200
Triglycerides	1.49	Mmol/l	1.7–2.2

**Table 2:** Blood investigations on Day 1, Day 2, and Day 3 of hospitalisation.

Labs	Day 1	Day 2	Day 3
Total white cell count	1.68	0.92	0.61
Absolute neutrophil count	0.98	0.59	0.25
Hemoglobin	7.3	6.1	5.2
Platelets	92	65	58
Ferritin	944		2536
Bilirubin	31		22
AST	41		83
ALT	19		53

## Additional Points

(1) A multidisciplinary approach by experienced clinicians including haematologists and infectious disease specialists is required in the management of the patient. (2) A combination of high fever unresponsive to broad-spectrum antibiotics, hyperferritinemia, hypertriglyceridemia, hypofibrinogenemia, cytopaenia, and organomegaly are key diagnostic clues. (3) Bone marrow biopsy should be obtained to confirm diagnosis. (4) Thalassaemia intermedia (HbH disease) classically has increased erythroid precursors on bone marrow examination. Pure red cell aplasia as demonstrated by rare giant erythroblasts with inclusion bodies on bone marrow examination is highly suggestive of a parvovirus B19 infection suppressing erythrocytosis. Parvovirus B19 serology should be done to confirm this if available.
